# A Guide to Christmas Benefactors

**Published:** 1920-12-18

**Authors:** 


### A GUIDE TO CHRISTMAS BENEFACTORS.

**At this Christmas-time, more than at any for many years past, our hospitals need the sympathy and practical support of all those who value and wish to perpetuate the Voluntary Hospital System, the influence of which is felt in every household in the Kingdom, through the training of medical men and nurses, the dissemination of knowledge brought to light by the study of medical, surgical, and hygienic methods of treating the sick, if not by direct help by hospitals to the individual. It is hoped that in no household will the great Christian Festival be allowed to pass without some thankoffering being sent to one or other of the hospitals of the United Kingdom.**

**ALL SAINTS' HOSPITAL, VAUXHALL BRIDGE ROAD, S.W.1.**—The estimated expenditure consequent upon the opening of the new in-patient department at 91 Finchley Road will be £8,000 a year, but the income from investments is only about £600 a year, the rest has to be made up by patients' fees and voluntary subscriptions. The Secretary is Mr. T. Mellor.

**BRITISH HOSPITAL FOR MOTHERS AND BABIES (NATIONAL TRAINING SCHOOL FOR DISTRICT MIDWIVES), WOOLWICH.**—This hospital trains midwives for work at home and abroad, and gives double the customary period of training in order to fit them for their responsible work. It is refusing twenty to thirty mothers a week for lack of room. The new enlarged building is now in hand, and a grant of £30,000 has been made by the Ministry of Health, but another £50,000 is needed in 1921 to complete the work. Donations and annual subscriptions are urgently requested from those who are interested in improving the work of midwives and so saving the lives of countless mothers and babies. Miss Alice S. Gregory is the Hon. Secretary.

**THE BELGRAVE HOSPITAL FOR CHILDREN, CLAPHAM ROAD, S.W. 9.**—This well-known hospital for children is in need of increased support. The cost of maintenance has increased from its pre-war figure of £4,500 to £9,000 per annum. It is the only children's hospital in South-West London, and is situated in the centre of a district teeming with children of the working classes. The present accommodation for in-patients—viz., forty cots—is not nearly sufficient to meet the needs of the district. The Committee are therefore anxious to complete the erection of the South Wing, which is already built up to the first floor. £10,000 is required for this purpose. A Building Extension Fund is now open, and contributions either to the Maintenance Fund or to the Building Fund will be most welcome. The Secretary is Mr. T. Clapham.

**CHELSEA HOSPITAL FOR WOMEN, ARTHUR STREET, CHELSEA, S.W.**—This special work for women is being crippled for want of adequate support and by the present high prices. It should appeal with special force not only to women for the less fortunate of their sex, but also to all who realise the importance of health and strength to the rising generation. It is submitted that the health of the women is the health of the children. £3,000 for the general maintenance fund is badly needed, and Mr. Sidney H. Goldsmid, the Hon. Treasurer, will gladly receive contributions at the hospital.

**CITY OF LONDON (CHEST) HOSPITAL, VICTORIA PARK, E. 2.**—Increased costs bring the expenditure this year to £33,000 and make the financial outlook serious. The hospital's work is of supreme importance owing to the increased ravages of consumption, of which the heavy death-rate is a dreadful evidence. The deficiency in accommodation generally for the treatment of the disease makes the hospital's efforts towards shouldering this burden in the East-End of enormous value. To enable it to continue the work unimpaired, with its beds numbering 175 and extensive out-patient department, further help is urgently needed. Contributions will be gladly received at the hospital or by Barclays Bank, Ltd., 54 Lombard Street, E.C. 3. Treasurer, Sir G. Wyatt Truscott, Bt.; Chairman, Sir A. Kaye Butterworth; Secretary, Mr. George Watts.

**CITY OF LONDON MATERNITY HOSPITAL, CITY ROAD, E.C.**—The urgent needs are: Improved accommodation for its out-patient department and its child welfare and consultative maternity centres—steps towards this provision are being taken, the estimated cost being £10,000, a part of which it is hoped to obtain by means of grants. A convalescent home (with ambulance service), by which means the number of admissions to the hospital could be doubled. The total debt now stands at £6,100.

**EVELINA HOSPITAL FOR CHILDREN, SOUTHWARK.**—The "Evelina," which is the largest children's hospital in South London, is in a desperate financial position. Over £5,000 is owing to the bankers, and the deficit at the end of the year will be not less than £6,000. Moreover, about £6,000 is required, also at once, thoroughly to renovate the hospital, which for many years, owing to shortage of funds, has had nothing beyond annual cleaning done to it. It is necessary to increase the annual income by at least £3,000 to meet the ordinary expenditure, which now amounts to £12,000 a year. Owing to its situation in the poorest district of South London, the "Evelina" is rarely seen by the well-to-do, and suffers accordingly, although it is carrying on as good a work as any Charity in London. Contributions for any amount will be thankfully received by Mr. H. C. Staniland Smith, the Secretary.

**EAST LONDON HOSPITAL FOR CHILDREN, SHADWELL, E. 1.**—The story of this hospital is similar to that of most of the other London hospitals. It is very much in debt—more than ever before. The Committee has been obliged to sell out securities, and unless substantial aid is forthcoming in the ensuing year there appears to be no alternative but to close the doors, which would be a disastrous thing for this neighbourhood. Mr. W. M. Wilcox is the Secretary, and he will gladly receive contributions.

**FLORENCE NIGHTINGALE HOSPITAL FOR GENTLEWOMEN, 19 LISSON GROVE, N.W. 1.**—Designed to meet the needs of the educated woman of small means on whom illness presses very heavily, especially in these days, it provides free medical and surgical treatment for the relations of clergy, naval, military, and professional men, artists, governesses, hospital nurses, etc. Fees, 30s. to £3 3s. per week. There is no endowment. A few investments bring in £150 per annum, and the fees equal about two-fifths of the expenditure, so that the balance has to be made up by subscriptions and donations. It is estimated that there will be a deficit of some £1,200 at the end of this year. The Chairman, Mrs. William Bridgeman, asks for Christmas gifts.

**GUY'S HOSPITAL.**—As a direct consequence of the war, the annual expenditure of the hospital has risen from £76,569 in 1914 to £129,901 in 1919, and it is only by the strictest and most vigilant economy that the governors have been able to meet this expenditure, and prevent the hospital from incurring debt. The governors of Guy's Hospital still believe that personal administration and individual devotion will best secure the efficiency and promote the scientific reputation of the hospitals, and in their present appeal for additional income they are searching for a way in which these may be maintained undamaged, preserving the spirit of philanthropy as well as zeal in the pursuit of knowledge and the welfare of humanity. Contributions should be sent to the Treasurer, Guy's Hospital, S.E.

**HAMPSTEAD GENERAL HOSPITAL, HAVERSTOCK HILL, N.W.**—The Hospital needs £22,000 annually to carry on its work. There are 128 beds. Ordinary sources do not yield more than £15,000. An urgent appeal is made for the deficit of £7,000. Contributions will be gratefully received by the Secretary.

**THE HOSPITAL FOR EPILEPSY AND PARALYSIS, MAIDA VALE.**—This institution stands in need of £1,000 to pay for repainting deferred during the war, and £10,000 to extend its out-patient department, which owing to insufficiency has almost become truly an outdoor department, because numbers of patients have to wait out of doors; this extension, whilst involving very little additional annual cost, will add enormously to the efficiency of the hospital. Mr. H. W. Burleigh, the Secretary, will gladly acknowledge the receipt of large or small gifts.

**KENSINGTON AND FULHAM GENERAL HOSPITAL, EARL'S COURT ROAD, LONDON, S.W. 5.**—This is the only general hospital in the boroughs of Kensington and Fulham. Patients treated in 1919 were 200 in-patients, 32,543 out-patients, of whom 1,337 were casualties. The hospital is entirely without endowment. Its income is derived from grants from the various hospital funds, subscriptions, donations, and entertainments, while small fees are paid by both in- and out-patients who have no subscribers' letters, though proved necessitous cases are treated free. The annual cost of upkeep is £4,500.

**KING'S COLLEGE HOSPITAL, DENMARK HILL, S.E.**—The Committee of Management earnestly appeal for substantial contributions either towards providing the £60,000 a year additional income so urgently needed or towards the several most pressing needs demanding large expenditure of a capital nature. The work of mercy and healing which is being carried on in King's College Hospital for the sick and helpless should appeal to all and impel them to give quickly, and above all to give generously, that this great work may be maintained and not in any way diminished. Those interested in the work of the hospital are invited to pay a visit to the institution, where they will find a hearty welcome extended to them at any time.

**THE LONDON HOMŒOPATHIC HOSPITAL, GREAT ORMOND STREET, W.C. 1.**—The Board of Management of the London Homœopathic Hospital, Great Ormond Street, W.C. 1, are making strenuous efforts to raise the sum of £7,690 before the close of the year for the following long-deferred and essential repairs: £1,831, renewal of electric-light installation; £1,103, renewal of boilers; £2,089, providing heating coils to warm domestics' dormitories, conversion of two lifts from hydraulic to electric power; £2,667, to complete appeal for £16,831 issued in 1916). King Edward's Hospital Fund for London have promised £1,250 if the whole sum is raised, and other good friends have generously donated. Unfortunately, however, the board's efforts are meeting with but a slow response, while to add to their difficulties there is every prospect that the hospital's accumulated deficit of £14,164 will assume even greater proportions with the close of the financial year.

**THE LONDON LOCK HOSPITAL AND HOME, HARROW ROAD, W.**—

I hear the voice of the unborn children,
The helpless children, night and day,
I hear it waking, I hear it sleeping,
It rings in my heart and I hear it say:
"What is this heritage you have given us,
You who should cherish us, loving and strong?
What have we done that your sin should visit us,
Dooming us, damning us, all life long?"

(The first verse of a poem written in aid of the Christmas appeal for the London Lock Hospital.)

**LONDON FEVER HOSPITAL, LIVERPOOL ROAD, N. 1.**—Over 2,700 sufferers of all ages were received here for treatment during the years 1917 to 1919. Typhoid, scarlet fever, diphtheria, spotted fever, measles, and German measles were the principal diseases. Expenses have been very heavy. Subscriptions and donations have fallen off, and additional help is earnestly appealed for.

**MOUNT VERNON HOSPITAL, NORTHWOOD, MIDDLESEX.**—The Mount Vernon Hospital is in need of additional help. In 1919 the total expenditure exceeded the total income by £4,854, and since then a further sum of £5,000 stock has been sold to repay a loan from the hospital bankers. The new building—begun in 1914, and upon which work was suspended during the war—still remains in an unfinished condition. In the present year the total expenditure is in excess of the total income, and new and increased help is urgently needed. The Secretary is Mr. W. J. Morton.

**MILDMAY MISSION HOSPITAL, BETHNAL GREEN.**—An immediate need is the sum of £340 to defray half the cost of the iron outside staircase now being erected to provide extra means of escape in the event of fire. The balance has been promised from the surplus Red Cross funds. A second urgent need is the installation of electric light over the entire hospital. The estimated cost is about £500-£600. With regard to current expenditure, the hospital is just able to keep its head above water, but it is hoped to close the financial year free of debt. A lean time, however, between January and April is anticipated. This can only be bridged by a considerably increased number of annual subscribers and donors. In the meantime a Christmas gift of £1,000 to pay for the staircase and electric light would be very acceptable.

**NATIONAL HOSPITAL FOR THE PARALYSED AND EPILEPTIC, QUEEN SQUARE, W.C.**—In its sore need a few months ago, when everything pointed to the National Hospital for the Paralysed and Epileptic, Queen Square, closing its doors for lack of funds, the hospital asked for the aid of patients, past and present, to get together part of the sum necessary to tide the institution over the anxious time. Many hundreds have come forward and taken boxes and collecting cards, and the harvest of small contributions is now commencing. One patient has collected £110 in six months, and many others are getting what in the aggregate are quite substantial sums. If the needs of this, the largest hospital for the treatment and study of nervous conditions, were thoroughly and widely known, it is felt that the £30,000 a year needed for maintenance would be easily raised. There are 267 beds, 50,000 attendances of out-patients, and 93 pensions for the incurable

**PRINCE OF WALES'S HOSPITAL, TOTTENHAM, N.**—£18,000 required annually. £7,000 in debt. No letters required. One hundred and twenty-five beds. Over 30,000 patients treated each year. Hospital situated in one of the most populated and poor districts in the Metropolis. Treasurer, the Right Hon. Sir George Murray, G.C.B., D.S.O. Director, Mr. F. W. Drewett, F.C.I.S.

**QUEEN CHARLOTTE'S LYING-IN HOSPITAL.**—Queen Charlotte's Hospital is in very great need of help both for maintenance and for urgent extensions and improvements, which had to be postponed during the war. The expenditure is quite double what it was before the war, and there is now a deficiency of over £10,000 on the income and expenditure account. The Committee are also appealing for £100,000 for extension and improvements which are greatly needed to bring the hospital abreast of modern requirements. The great increase in the amount of operative midwifery urgently calls for the provision of an operating theatre. Difficult cases of labour are received from all parts of the country, and with the development of the schemes of maternity and child welfare the demands on the hospital are daily increasing. Contributions are earnestly requested, and they may be sent to the Secretary (Mr. Arthur Watts) at the Hospital, Marylebone Road, N.W. 1.

**THE ROYAL WESTMINSTER OPHTHALMIC HOSPITAL.**—Greatly in need of funds. For 104 years the institution has relieved sufferers from injuries and diseases of the eye. Nearly 40,000 patients attend for treatment annually, coming not only from the Metropolis, but from all parts of the country. Will all those blessed with good eyesight kindly remember the unfortunate sufferers this hospital helps relieve and in many cases saves from blindness. Surely there can hardly be a more terrible calamity! Contributions either in money or in kind will be gratefully acknowledged by the Secretary, at the Hospital, King William Street, West Strand, W.C. 2.

**THE ROYAL NATIONAL ORTHOPÆDIC HOSPITAL, GREAT PORTLAND STREET, LONDON, W.**—What does "Orthopædic" mean? Making crooked children straight. The great majority of the patients are children. Orthopædic surgery is concerned with the prevention and cure of deformity. The number of children who, through neglect in their early years, grow up crippled with club feet, crooked limbs, etc., is appalling. Lord Denbigh, the Chairman, asks, on behalf of the Appeal Committee, for contributions towards this national work.

**THE ROYAL DENTAL HOSPITAL, LEICESTER SQUARE, W.C.**—The Royal Dental Hospital has now been established sixty-two years, during which time an incalculable amount of suffering has been relieved and many serious illnesses averted by timely attention to the teeth of the poorer classes. The Committee of Management wishes to make a special appeal to extinguish the debt of £22,680 on the building, which so seriously cripples the funds of the charity. After sixty-two years of hard work, it is earnestly hoped that this truly national institution may be freed this year from the burden under which it is still struggling. Contributions may be sent to the Secretary.

**ROYAL FREE HOSPITAL, GRAY'S INN ROAD, W.C.**—The financial position at the close of this year is critical—expenditure having exceeded the income by upwards of £25,000. Although there has been increased income, this has not balanced expenditure. The reserve funds available for meeting deficits in income are now exhausted, and it is feared some curtailment of the work must be considered unless means are found for meeting the expenditure. The estimated income for 1921 is not more than £20,000, while the expenditure cannot be estimated at less than £55,000. A scheme has been prepared for an extension of the hospital to provide accommodation and treatment of patients, who will contribute the whole cost of maintenance, but progress cannot be made in this direction until satisfactory arrangements are made for general maintenance of the existing beds. The Secretary is Mr. Reginald Garratt.

**ST. MARY'S HOSPITAL FOR WOMEN AND CHILDREN, PLAISTOW, E. 13.**—The position of this hospital is most serious. The heavy increase in maintenance during the past two years, and the little support received from the public, has rendered it necessary for the Committee to sell the whole of the general fund investments to pay off the overdraft at the bank and loans. The hospital has now no reserve, and it is anticipated that at the end of the year a new debt of about £400 will have to be met. The matter is so serious that a special meeting of the Management Committee is to be held on December 20 to consider reports from the Medical Committee, etc., as to what work of the hospital can be discontinued at once. Unless very substantial help is forthcoming during the present month the closing down of the hospital must commence early in the new year.

**ST. SAVIOUR'S HOSPITAL FOR LADIES OF LIMITED MEANS, OSNABURGH STREET, N.W. 1.**—There is hardly a class of patients that merits public sympathy more, and perhaps none for whom less provision is made, than that of poor ladies. The gratitude of those who are benefited by this charity is very real and often extremely touching, and daily experience at St. Saviour's gives continuous proof of how great is the need that the hospital was designed to meet. The small payments made by patients cannot cover expenses, and there is no endowment, so the hospital is largely dependent upon voluntary support, which is earnestly asked for. Subscriptions and donations may be sent to Sir Cyril Cobb, K.B.E., M.V.O., M.P., at the hospital.

**ST. MARK'S HOSPITAL, CITY ROAD.**—In pre-war days the annual expenditure hardly exceeded £5,000; to-day £12,000 is required. The sum of £7,000 is urgently required to help carry on the work and to pay off a banker's loan of £1,500. In 1914 it was decided to carry out improvements and extensions to the nurses' quarters that were even then overdue. For this a sum of £2,000 is needed at once, and towards this sum the King's Fund have promised £600, provided that a similar sum can be obtained before March next. Mr. H. Coope, the Secretary, will answer all inquiries.

**ST. JOHN'S HOSPITAL FOR DISEASES OF THE SKIN, LEICESTER SQUARE, W.C. 2.**—This Hospital, founded in 1863, is the largest of its kind in the kingdom, dealing with 1,000 cases a week. People are apt to regard skin diseases as trivial and beneath notice, or of being due to preventable causes, and thus the fault to a large extent of the sufferers themselves. Such persons are invited to visit the hospital to see and judge for themselves at first hand as to the seriousness and the pathos of skin diseases. There is no risk whatever that visitors may contract infection, as many seem to think. As to their being preventable, the same may be said of other diseases, and especially of accidents. The wards have been recently decorated under the Kemp Prosser scheme of colour, and are very bright and cheerful. Like other institutions, this hospital is suffering from the present high prices of all commodities, and it is feared that the close of the year will show a serious deficit. To turn away the patients and to close down would inflict great hardships. The Board of Management appeal earnestly to those in fortunate circumstances to open their hearts to the needs of this thoroughly deserving and necessary institution. Cheques would be gratefully received by the President and Treasurer, the Earl of Chesterfield, K.G., 49 Leicester Square, W.C. 2, or by the Secretary, at the same address.

**ST. JOHN'S HOSPITAL, LEWISHAM.**—Financial support is needed by this hospital because (1) with the exception of the South Eastern Hospital for Children at Sydenham, it is the only voluntary hospital in the large borough of Lewisham. (2) The number of in-patients increased from 328 in 1910 to 626 in 1919, and the number of out-patients and attendances of them increased from 332 and 1,390 respectively in 1910 to 4,075 and 26,688 respectively in 1919. (3) Upon the raising of £1,000 depends the receiving of the promised contribution of £1,000 towards the expense of providing the extended Out-patients Department. When £1,000 means £2,000, he who gives quickly gives twice, and the Hon. Treasurer will be glad to hear from him.

**ST. MARY'S HOSPITAL, PADDINGTON, W. 2.**—St. Mary's Hospital is the principal general hospital for the West and North-West districts of London, which contain a population of over half a million. It provides annually for 4,000 in-patients and 40,000 out-patients. Throughout the war it received sick and wounded soldiers from the Front, and supplied anti-typhoid and other vaccines for the Allied Armies, by which thousands of lives were saved. To carry on the work efficiently, in present conditions, costs about £70,000 a year. Contributions may be sent to the Treasurer, or the Secretary, at the hospital.

**SAMARITAN FREE HOSPITAL FOR WOMEN, MARYLEBONE ROAD, N.W. 1.**—The Committee of the Samaritan Free Hospital for Women, Marylebone Road, London, earnestly appeal to readers of The Hospital, especially to women, for assistance to enable them to carry on. The most pressing need is money to prevent closing some of the small wards. The whole of the electric-light installation has been condemned and its renovation is imperative. Pressing though this is, the continuance of the full complement of beds is much more so. The waiting list now exceeds 150, and to close even a single bed means additional suffering, a longer waiting period, and a heavier list. The loss in annual subscriptions this year alone exceeds £100. Will some kind friend make this good? Any contribution, however small, addressed to the Chairman will be most gladly welcomed and thankfully received.

**THE SOUTH LONDON HOSPITAL FOR WOMEN, CLAPHAM COMMON.**—This is a general hospital for women and children, officered by women doctors. This hospital has a very special need at the present time—namely, the erection of an out-patient department. The work is now being carried on in adapted premises, which are totally inadequate, with the regrettable result that during 1919 no fewer than 4,613 women had to be turned away. A freehold site has been placed at the disposal of the hospital, and it is earnestly hoped that sufficient support may be forthcoming to enable this necessary work to be begun without delay. Under present conditions it is estimated that a sum of £50,000 will be required to meet the cost of building and equipment. Additional support is urgently required to meet the heavy expenses of increased salaries and wages, food, fuel, and drugs. The Board of Management earnestly appeal for help.

**UNIVERSITY COLLEGE HOSPITAL, GOWER STREET, W.C.**—The financial position, in common with many other London voluntary hospitals, is reaching an acute stage. The deficit for the year 1919 was nearly £12,000, and the prospective deficit for the current year is estimated at about £20,000. The borrowing power of the hospital will be exhausted before the end of the year, and the problem of carrying on the work of the hospital next year is causing the greatest anxiety to the board of management. In-patients are being asked to contribute towards their maintenance on a voluntary basis in proportion to their means, and out-patients are encouraged to contribute towards their medicines, dressings, and special treatment, but the maximum revenue obtainable from these sources is hardly likely to cover more than one-tenth of the expenditure. The need of increased public voluntary support cannot be too strongly urged.

**THE VICTORIA HOSPITAL, TITE STREET, CHELSEA.**—This well-known hospital for children is very much in need of additional financial help to enable the Committee to meet the greatly increased cost of maintaining the work. The current year began with a deficit of £3,663, which has since increased to over £5,000. Unless further financial help is received, this debt will go on increasing until all the Committee's available resources are exhausted, and the only course left will be to close the hospital. In order to maintain the good work that is being done for the children, an additional £6,000 is required to the annual income, and £20,000 is urgently needed for necessary improvements. Mr. H. G. Evered, the Secretary, will gladly receive Christmas gifts.

**THE WESTERN OPHTHALMIC HOSPITAL, MARYLEBONE ROAD, N.W. 1.**—A freehold house in Marylebone Road, used, so the legend runs, by George III. as a country convalescent house for his children, contains some fine specimens of Adam chimney-pieces, but its roof has perished and badly leaks. A few weeks since a ceiling (hundredweights of massive plaster) fell down. Such a happening would doubtless have caused glee amongst the Royal children, but the house for the past sixty-four years has been used as an eye hospital, and this particular ceiling was in the operating theatre. One shudders whilst thinking of what would have happened had an operation been in progress, with some poor soul lying on the operation table, wide eyed, facing the ceiling, and full of trust in the surgeon, who might have been standing by the table with his scalpel poised over the eye. If you were a member of the Hospital Committee, would you vote to shut the hospital (it is not in debt, and can maintain its work), and cut off treatment for 10,000 patients annually, or would you accept responsibility for the risk of housing patients in such a building until rebuilding can be effected? If the latter, would you help to raise the £50,000 needed, or are you a potential fairy, who could by the wave of a pen at once make rebuilding possible? The scheme is admirably adapted for a memorial. H. W. Burleigh is the Honorary Secretary.

**WESTMINSTER HOSPITAL, S.W.**—This institution is in urgent need of help. During the current year strenuous efforts have been made to increase its income. The expenditure for this year will amount to upwards of £45,000, and it is feared that it will be even higher in 1921.

**DR. BARNARDO'S HOMES.**—The Charter of Dr. Barnardo's Home is that "no destitute child is ever refused admission." Children are received daily from the wretched slums and alleys of our great cities. Of such children as these were made the Barnardo heroes of the great war, and children such as these can, if properly trained, help to move the world forward in the right direction.

